# Inclusion of a Parental Component in a Sports-Based HIV Prevention Program for Dominican Youth

**DOI:** 10.3390/ijerph20126141

**Published:** 2023-06-16

**Authors:** Chrys Gesualdo, Helle Larsen, Pilar Garcia

**Affiliations:** 1 Department of Developmental Psychology, Philipps University, 35032 Marburg, Germany; 2 Department of Developmental Psychology, University of Amsterdam, 1018 WS Amsterdam, The Netherlands; 3 Department of Educational and Developmental Psychology, University of Valencia, 46010 Valencia, Spain

**Keywords:** HIV/AIDS, health, parenting, adolescent sexual behavior, self-efficacy

## Abstract

Background: Underprivileged youth in the Dominican Republic (DR) are at high risk of acquiring the human immunodeficiency virus (HIV). Protective parenting practices may inhibit sexual risk-taking. Objective: We investigated whether parental involvement in a sports-based HIV prevention program increased self-efficacy to prevent HIV and safe sex behavior among Dominican youth. Method: The study had a quasi-experimental design with repeated measures. *N* = 90 participants between 13 and 24 years of age participated in the program through two different trainings, UNICA and A Ganar, both of which had an experimental (i.e., program with parental component) and a control (i.e., program without parental component) condition. Results: Self-efficacy to prevent HIV significantly increased among participants in the experimental condition of UNICA. Self-efficacy for safe sex increased among sexually active participants in the experimental condition of A Ganar. Implications for Impact: These findings are important to meet the United Nations’ Sustainable Development Goal of good health and wellbeing, as they suggest that parental involvement in sports-based HIV prevention programs can enhance their positive effects for increasing youth’s self-efficacy to practice HIV-preventive behaviors. Randomized control trials and longitudinal studies are needed.

## 1. Inclusion of Parents in a Sports-Based HIV Prevention Program for Dominican Youth

The island of Hispaniola, which is composed by the Dominican Republic (DR) and Haiti, encompasses the highest rate of human immunodeficiency virus (HIV) and acquired immune deficiency syndrome (AIDS) infections in the Caribbean [1], as a high amount of infections are reported per year in the DR [2]. A high prevalence of HIV among Dominican youth between 15 to 24 years has been reported, as this age group has a higher risk of acquiring sexually transmitted infections (STIs) and HIV than the rest of the population [3]. Rural areas and leading regions for sex tourism have the highest prevalence of HIV in the country [4]. Some of the leading factors contributing to this prevalence are a lack of adequate sexual education resources for rural populations [5] and risky adolescent sexual behavior [3]. Risky sexual behavior is described as having unprotected intercourse, inconsistent use of birth control, having several sexual partners, changing sexual partners frequently, and having an early sexual debut [6]. A high percentage of Dominican youth between 15- and 24-years report having had sex without condoms, having had more than one sexual partner, and having had sexual intercourse before the age of 15 [2,3].

Although sexual education is part of the curriculum of primary and secondary schools in the DR, youth are not thoroughly instructed about preventive behaviors [7] and, as a result, a large percentage of Dominicans do not possess comprehensive knowledge of HIV [4]. Evidence consistently supports the notion that parents highly influence their adolescent’s sexual decisions [8]. The influence of parents in their youth’s sexual development is particularly relevant for the youth of Hispanic heritage, whose cultural values place an imperative focus on family interactions [9]. However, they are not provided with sufficient trainings nor practical support or practices to counteract risky behavior in youth [10]. Furthermore, parents often show difficulties in enacting protective parenting practices [7].

Parent–child communication about sex has been linked to safer sexual decision-making in youth [8]. A study showed that the role of parent–child sexual communication might be mediated by the parent–child quality of relationship, perceived support of parents, and the content and quality of the talk [8]. If equipped with tools to become better communicators and provided with comprehensive knowledge about healthy sexual practices, parents could be an invaluable resource to promote sexual health in their youth [11]. Important topics include conversations about birth control, what is right and wrong, delaying sexual debut, sexual decline negotiation skills, clear rule setting, and communicating love and support [8].

Another core mechanism through which parents can modify risky sexual behavior is monitoring, which is characterized by knowing their child’s whereabouts, peers, and activities, as well as enforcement of rules about dating [12]. High levels of overall parental monitoring and rule enforcement were related to delayed initiation of sexual activity and greater condom and contraceptive use [12]. Longitudinal studies show that perceived parental monitoring was predictive of increased condom use among adolescents at 12-, 16- and 36-months since a follow-up [13]. Thus, sexual health interventions that promote parental monitoring are more likely to decrease adolescent risky sexual behavior than those that do not target this practice [14].

A good-quality relationship is regarded as one involving affective connection, positive interactions, and constructive communication [15]. Youth who have a high-quality relationship with their parents have a later sexual debut [16], practice safer sex [17], and have less sexual partners [18] than youth who have a poor-quality relationship with their parents.

Although protective parenting practices align with decreased adolescent risky sexual activity, adolescent self-efficacy to practice HIV preventive behaviors is a mediating factor that contributes to the likelihood of engaging in safe sex [19]. Social cognitive theory (SCT) provides a framework for how personal characteristics (e.g., self-efficacy to practice safe sex and HIV preventive behaviors) and environmental factors (e.g., communication with parents, parental monitoring, and relationships with parents) influence an agent’s (e.g., youth’s) behavior (e.g., safe sexual behavior) [20]. Self-efficacy beliefs can predict the enactment of desired behavior, motivation to perform, and perseverance through adversity [20]. Yet, this is highly influenced by an encouraging and supportive environment [20]. By leveraging observational learning (parental involvement can serve as a role-modeling mechanism, thereby promoting the adoption of effective strategies and enhancing self-efficacy beliefs in youth), vicarious experiences (youth can gain confidence and develop self-efficacy beliefs by learning from their parents’ successful implementation of preventive behaviors), social persuasion (parental encouragement, support, and effective communication can enhance youth’s self-efficacy by instilling confidence and reinforcing positive beliefs about their abilities to engage in HIV-preventive behaviors), and mastery experiences (through parental involvement, youth can engage in skill-building activities, such as communication exercises and role-playing scenarios with their parents, which allow youth to practice and refine their skills related to HIV prevention and safe sex, thereby leading to increased self-efficacy), parental involvement can enhance a youth’s self-efficacy to prevent HIV and engage in safe sex practices. Thus, increasing adolescent self-efficacy to practice safe sex by simultaneously promoting a protective familial environment is a critical strategy for sexual health interventions.

### 1.1. Sexual Health Interventions for Youth

The most-used method of sexual health education is school-based [21] and often promotes abstinence only [22]. Several interventions have been implemented in school settings that are complimentary to mandatory sexual health education to further educate youth about preventive sexual behaviors [21,23,24]. Despite encouraging trends signaling their efficacy at improving sexual health knowledge, their effect on behavior is low [22].

The scant research to date about sports-based HIV/AIDS prevention programs supports their efficacy at promoting healthy sexual decision-making in underprivileged youth outside the school setting [25,26]. Their aim is to encourage sexual health and to promote positive behavior change by increasing knowledge and skills through educational soccer activities taught by trained local role models [27,28]. Evidence supporting the efficacy of peer-led interventions to reduce HIV risk is mixed, with findings indicating increased sexual health knowledge [29,30,31,32] and condom use intentions [31,32], while showing inconsistent results on behavior [29,31,32]. In addition, a meta-analysis on parent-based sexual health interventions reported medium to large positive effects regardless of delivery dose (i.e., time, frequency, duration) [33]. Furthermore, family-level interventions can deliver more positive results than traditional school-based strategies [34].

Deportes Para La Vida (DPV) is a sports-based HIV/AIDS prevention program targeting at-risk youth aged 13 to 24 [35]. DPV is delivered by the Dominican Republic Education and Mentoring (DREAM) Project, which is a non-profit organization that provides life-skills development opportunities to resource-constrained youth throughout the DR [36]. DREAM is located in Puerto Plata, which is a province with several impoverished communities implicated in sex tourism. DPV is based on the methodologies of Grassroot Soccer [30]. The program uses sports-based activities, games, and interactive sessions to provide training in which young people learn about the risks of sex, the myths surrounding STIs and their transmission, and gender equity. DPV’s efficacy at increasing HIV-related knowledge among participants in an experimental compared to a control group has been demonstrated [2,25]. Empirical research has yet to investigate DPV’s effect on behavior.

DREAM delivers the DPV curriculum through two different programs, namely, ‘UNICA’ and ‘A Ganar’. The content in terms of sports activities of the DPV intervention is the same in both programs. However, the target sex, age group, and dose differ. UNICA targets females between 13 and 17 years and is delivered at a low dose (i.e., twice a week, two hours per session, for four weeks) [35]. A Ganar is a professional-skills building program targeted at 18- to 24-year-old males and females that begins with an intense dose of DPV (five days a week, 2 h per session, for eight days) [35]. Currently, DPV targets adolescents only, without considering their environment. Given that interventions targeting several behavior-influencing factors (e.g., parents) have been associated with more positive outcomes, the inclusion of a parental component to DPV as an additional preventive measure can further promote adolescent healthy decision making.

### 1.2. The Present Study

The primary aim of this study was to investigate the effect of including a parental component to DPV in two self-reported adolescent outcomes: (a) self-efficacy to prevent STI’s/HIV and (b) safe sexual behavior among already sexually active youth. We applied the principles of SCT to develop a parental intervention for inclusion to DPV in which strategies to enhance parent–child sexual communication, parental monitoring, and parent–child relationship quality were provided. We hypothesized significantly higher self-efficacy to prevent STI’s/HIV among participants in the experimental condition (i.e., DPV with parental component) than in participants in the control condition (i.e., standard DPV without parental component). In addition, we hypothesized significantly safer sexual behavior among sexually active participants in the experimental condition than in sexually active participants in the control condition. Furthermore, we explored whether the delivery intensity of DPV (i.e., UNICA at a low dose and A Ganar at a high dose) influenced the effectiveness of the parental component at increasing STI/HIV-preventive behaviors among participants in the experimental conditions of both programs.

## 2. Method

Our study had a quasi-experimental design with repeated measures on a control and experimental group [37]. Parents of all DPV participants were invited to a workshop. Participants whose parents attended the workshop were included in the experimental condition, while those whose parents did not attend were included in the control condition. To address potential contamination, participants in the control and experimental groups received the intervention separately. Moreover, the importance of confidentiality was emphasized, and participants in the control condition were asked not to share information or experiences with others. To explore the influence of DPV dose on the effectiveness of the parental component, participants of different age groups received equal interventions at different doses separately through UNICA and A Ganar.

### 2.1. Participants

**Figure 1:**
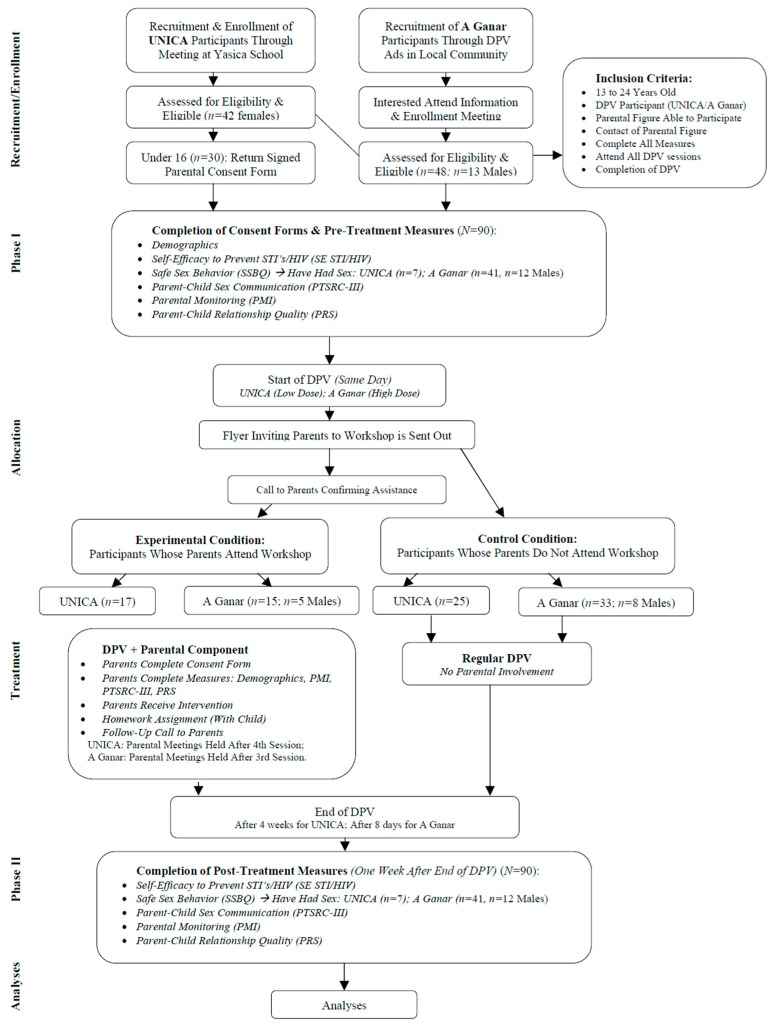
Study flow diagram.

Inclusion criteria were being between 13 to 24 years old, participation in DPV through UNICA or A Ganar, having a parental figure and being in contact with them, willingness to complete all measures, attendance to all sessions, and the completion of DPV (see Figure 1). Inclusion criteria for parental figures required their participation consent, willingness to provide contact information, attendance of a workshop, and completion of a homework assignment.

*UNICA.* To recruit participants, a DPV team member informed 9th and 10th grade students about the study before the start of the program. Those under the age of 16 were given a parental consent form for parents to sign and were asked to return it prior to the start of UNICA to participate in the study. *N =* 42 female students aged 13 to 17 participated in UNICA. The experimental condition was composed of *n* = 17 participants (*M*_age_ = 15.29 years, *SD* = 1.21), out of which *n* = 3 reported having had sex.

*A Ganar.* DREAM advertised the beginning of a new cycle of A Ganar in local establishments. An information meeting was conducted prior to the start of the program in which a DPV representative introduced the study and recruited participants. A total of *n* = 48 participants (*n* = 13 males) aged 18 to 24 participated in A Ganar. The experimental condition consisted of *n* = 15 participants (*n* = 5 males; *M*_age_ = 19.13 years, *SD =* 1.13), out of which *n* = 12 (*n* = 5 males) stated having had sex.

### 2.2. Procedure

Ethical approval was granted by the Ethics Review Board at the University of Amsterdam, Netherlands. Personal informed consents from students above 16 years and initial data collection occurred on the first day of DPV before initiating the program. Flyers inviting a parental figure to the workshop were sent out during the first week of DPV, and participants were asked to confirm parental attendance. We did not administer post-tests to parents, as we expected to see the effects of increased parental competence to apply protective parenting practices reflected in their adolescent’s self-efficacy to practice safe sex. For UNICA, the parental workshop took place after the fourth session of DPV, and for A Ganar after the third session. All participants completed the post-tests a week after completing DPV.

Parental Component for DPV. To design the intervention, we employed the iterative steps from the intervention mapping (IM) protocol [38]. IM is a planning tool for the development of theory-and-evidence-based health promotion interventions using an ecological approach [38]. The parental intervention was delivered by a Development and Health Psychology graduate student and consisted of a workshop, as well as a homework assignment, to complete with their children (see Figure 1). The workshop and assignment were partly designed after the parent-based *Families Talking Together* intervention, as it has shown positive effects at reducing adolescent sexual risk behavior, delaying the initiation of sexual activity, and decreasing the frequency of sexual intercourse [39]. Homework assignments can facilitate the parent–child sex talk and provide a structured opportunity for this type of conversation to arise [40]. Furthermore, research suggests that adolescents would like to hear and discuss more of their parent’s sexual experiences when they were their age, as they regard this type of communication as memorable and impactful [41]. Thus, the assignment consisted of two exercises and provided the opportunity to address various sexual topics.

Upon arrival to the workshop, parents completed informed consent forms and the questionnaire. Based on the protocol of the *Families Talking Together* intervention [39], as well as on practical constraints and feasibility of implementation, the workshop lasted 60 min (parents attended the total duration). The workshop informed parents about general communication, relational, and monitoring skills [39], as well as about practices to adopt to satisfy what adolescents have reported to seek in interactions with their parents, such as a comfortable environment, less lecturing, more comprehensive and informative talks, being listened to, and feeling support [41]. To mimic the interactive educational style of DPV and to provide an environment where parents could reflect on their own practices, intentions to improve, and practice the skills covered, we concluded the workshop with a role-play activity. Volunteers acted the proposed behaviors for the audience to provide feedback, share thoughts, and express what they would improve in the interaction. Finally, parents received a summary of the material covered in the workshop along with the homework assignment. Parents were called three days after the intervention to ensure they worked on the assignment and practiced the learnings from the workshop.

### 2.3. Materials

Five validated self-report measures were administered to all participants at pre- and post-DPV (see Figure 1). For translation of the instruments to Spanish, the recommendations of Foster and Martinez were employed [42]. In the case that a student did not have parents, they were asked to answer with their parental figure(s) in mind. Anonymous coding was used to ensure participants’ privacy.

*Demographic Questions.* Age, sex, who do they live with, education, community where they live, closeness to parents, ease to remember the sex talk with parents, and whether they have had sexual intercourse or not.

*Self-Efficacy to Prevent STI/HIV*. The Self-Efficacy for STI’s and HIV Prevention Scale (SE STI/HIV), a 10-item scale in a Likert scale response format (1 = *not sure at all* to 5 = *completely sure*), was administered to assess self-efficacy to practice STI and HIV prevention behaviors (i.e., abstinence, loyalty to one partner, proper condom use, avoidance of alcohol and drugs, and sexual health communication with others) [43]. Scores ranged from 10 to 50, and higher scores denoted better perceived capacity for STI and HIV preventive behaviors. An internal reliability analysis on our data resulted in a Cronbach’s coefficient of 0.53.

*Safe Sexual Behavior*. The Safe Sex Behavior Questionnaire (SSBQ), a 24-item questionnaire with a 4-point Likert scale (1 = *never* to 4 = *always*), was administered among participants who stated that they have had sex (*n* = 7 in UNICA; *n* = 41 (*n* = 12 males) in A Ganar) to assess frequency of sexual practices that prevent or promote HIV (i.e., condom use, exposure to body fluids, homosexual practices, risky behavior, anal practices, and sexual communication skills) [44]. Scores ranged from 24 to 96, and higher scores indicated safer sexual practices. The Cronbach’s alpha for SSBQ found in our study was α = 0.66.

*Parent–Child Communication About Sex*. The Parent–Teen Sexual Risk Communication Scale (PTSRC-III) was administered to measure the frequency, content, and amount of communication about sexual topics (i.e., birth control, preventing STIs and HIV, sexual debut postponement, and resisting peer and sexual pressure) occurring between parents and their adolescents [19]. The PTSRC-III is an 8-item measure with a 5-point Likert scale response format (from 1 = *nothing* to 5 = *everything*). Scores ranged from 8 to 40, and higher scores represented extensive parent–child sex communication. The PTSRC-III showed excellent reliability in our data (α = 0.82).

*Parental Monitoring*. As a measure of parental monitoring from the adolescent’s perspective, the Parental Monitoring Instrument (PMI) was administered [45]. The PMI consists of 27 items rated in a Likert scale, ranging from 1 = *0 times* to 4 = *5 or more times*, and contains seven subscales representing monitoring strategies: ‘Direct monitoring’, ‘Indirect monitoring’, ‘School monitoring’, ‘Health monitoring’, ‘Computer monitoring’, ‘’Home monitoring,’ and ‘Restrictive monitoring’. Scores ranged from 27 to 108, and higher scores indicated more frequent monitoring. Cronbach’s Alphas ranging between 0.64 and 0.80 were found for all factors of the PMI on our data.

*Perceived Quality of Relationship with Parents*. Positive parent–child relationships were measured in response to the Positive Relationship with Parents Scale (PRS) developed by Child Trends for the Templeton Foundation, as part of the Flourishing Children Project [15]. The adolescent version of this scale consists of 6 items rating their perceptions of their relationships with their parent(s) (i.e., connectedness, support, positive interactions) using a 5-point Likert scale ranging from 0 = *none of the time* to 4 = *all of the time*. Scores ranged from 0 to 24, and higher scores indicated better perception of quality of relationship with parents. An internal reliability analysis of PRS for our study resulted in α = 0.80.

### 2.4. Data Analyses

Data analysis was conducted using SPSS Statistics^®^ version 25.0 (IBM Corp, Armonk, NY, USA). The dependent variables, self-efficacy to prevent STI’s/HIV and safe sex behavior, were continuous and normally distributed, as indicated by a Kolmogorov–Smirnov test. Independent *t*-tests and cross tab analyses were used to examine baseline differences between conditions regarding demographic variables. The first hypothesis was tested using repeated measures of an ANCOVA, including program and demographic variables as covariates and ‘condition’ as the between variable. To investigate our second hypothesis, an ANCOVA was performed whilst controlling for total composite baseline scores of SSBQ and demographic variables (i.e., age and sex) on post total composite scores of SSBQ with ‘condition’ as a fixed factor. To explore whether DPV’s delivery dose influenced the effects of the parental component, a repeated measures ANCOVA was conducted examining the interaction between conditions, dose (i.e., program), and time, including demographic variables as covariates. Pairwise comparisons were made to determine main differences.

## 3. Results

Sample characteristics are presented in Table 1 by dose (i.e., program) and condition. Participants of both programs were predominantly female, Dominican, and were receiving or had received a high school education. No significant differences between conditions for demographic variables in UNICA and A Ganar participants were found. Baseline and post-DPV mean scores by condition based on minimum and maximum scores of all measures are presented in Table 2.

### 3.1. Self-Efficacy to Prevent STIs/HIV

*UNICA.* Significant differences between pre- and post-SE STI/HIV scores between participants in the experimental and control conditions [*F*(1,37) = 4.22, *p* = 0.05] were found. Post hoc analyses indicated that participants in the experimental condition presented significantly higher scores in the self-efficacy to prevent STIs/HIV scale (*M* = 40.48, *SD* = 1.16) than participants in the control condition (*M* = 37.25, *SD* = 0.94).

*A Ganar.* No significant differences between pre- and post-SE STI/HIV scores were found between participants in the experimental and control conditions [*F*(1,40) = 1.31, *p* = 0.26].

### 3.2. Safe Sex Behavior

*UNICA.* No significant effects of condition after controlling for pre-SSBQ scores and demographic variables on post-SSBQ scores [*F*(1,1) = 11.88, *p* = 0.18] were found.

*A Ganar*. A significant effect of condition on post-SSBQ whilst controlling for baseline SSBQ scores and demographic variables [*F*(1,32) = 13.29, *p* = 0.00] was found. Participants in the experimental condition showed significantly higher safe sex behavior scores (*M* = 83.64, *SD* = 1.92) than participants in the control condition (*M* = 75.12, *SD* = 1.19).

### 3.3. Influence of Intervention Dose on Effect of Parental Component

A significant interaction effect between *Condition* × *Dose* × *Time* [*F*(2,85), = 12.45, *p* = 0.00] was found. Participants in the experimental conditions of UNICA and A Ganar showed significant improvements from the baseline (*M_UNICA_* = 38.22, *SD* = 1.72; *M_A Ganar_* = 29.65, *SD* = 1.67) to post-SE STI/HIV scores (*M_UNICA_* = 44.67, *SD* = 1.72; *M_A Ganar_* = 40.51, *SD* = 1.67), with a *p* = 0.04. Univariate testes indicated a significant effect of condition [*F*(3,85) = 3.21, *p* = 0.03]. Furthermore, multivariate effects of time were also found [*F*(1,85) = 69.11, *p* = 0.00]. A marginally significant effect of dose was found [*F*(1,85) = 3.57, *p* = 0.06].

## 4. Discussion

The present study drew on SCT to investigate the role of participant’s self-efficacy to practice safe sex and its association to protective parenting practices. In line with our first hypothesis, we found a significant difference in improved self-efficacy to practice STI/HIV preventive behaviors between participants in the experimental and control conditions of UNICA. The improvement was observed in participants in the experimental condition, which suggests an influence of parental involvement in the enhancement of SE STI/HIV. This result is consistent with previous studies investigating parental involvement in sexual health interventions reporting their effectiveness at promoting self-efficacy to practice safe sex in youth (e.g., [34,39]), and provides the novel notion that these positive effects may also be applicable for sport-based sexual health interventions. This outcome provides novel findings in the topic of parental involvement in sports-based sexual health interventions in the DR, given that no reports investigating this topic are available. Nonetheless, the observed low internal consistency imposed by the SE STI/HIV scale [43] raises concerns about the scale’s reliability, and caution should be exercised in interpreting the findings. While the scale demonstrates promising content validity, future research should employ additional measures to establish stronger criterion-related and to construct validity of the self-efficacy assessments in the context of STI and HIV prevention. Conversely, our first hypothesis was not supported for the A Ganar group, as no significant differences between conditions were found. This non-significant result can be related to a lack of sufficient statistical power. A power analyses indicated a required minimum of seventeen participants per condition. Due to limited parental availability, the experimental condition of A Ganar was composed of only fifteen participants. We controlled for this by providing flexible dates for the parental intervention to facilitate attendance; however, we could not control for actual disposition to attend. Moreover, we found a low internal reliability for the SE STI/HIV scale (i.e., α = 0.53) that may have influenced this difference in results. The difference in results between UNICA and A Ganar groups might also be related to the sex, age, dose and motives of participants. First, evidence suggests that girls generally communicate more with parents [46] and receive more monitoring than boys [46,47]. Being that the UNICA participants were all female, they may have been more receptive of the enhanced parenting practices and, thus, displayed better results than the A Ganar group. However, other researchers have not found sex differences in relation to the effect of protective parenting practices on risky sexual behavior [46]. Moreover, protective parenting practices may be particularly favorable at promoting self-efficacy for safe sex behavior if applied during early ages [19]. Similarly, the absence of parent–child communication about sexual topics and a positive parent–child relationship during early adolescence are associated with a premature sexual debut [8]. Therefore, the positive results of the parental component at increasing self-efficacy for safe sex in UNICA might be related to their younger age. A Ganar participants were older and, therefore, perhaps more independent and less influenced by parents. Thus, subsequent replications of our study should focus on recruiting a sample with a homogeneous sex and age distribution.

Furthermore, our findings did not support our second hypothesis for UNICA, given that no significant differences between conditions were found. It is important to note that the number of participants who reported being sexually active was relatively small (i.e., 7 out of 42). As such, our result might be inconsistent with the literature indicating that parental participation in sexual-health interventions may decrease risky sexual practices in youth [39] due to a lack of participants with sexual experience, and not due to the absence of an effect. Regarding A Ganar, we found evidence supporting our second hypothesis, as there were significant differences between conditions. As opposed to UNICA, the majority of A Ganar participants (i.e., 41 out of 48) indicated having had sex in their lifetime. The difference in ages between the A Ganar group and the UNICA group may have influenced the disparity of results between groups. As in UNICA, the sexually experienced and unexperienced statuses were not uniformly represented in order to test differences between conditions and determine generalizable results. While the direction of our analyses was in accordance with our research quests, based on our results, we are not able to draw conclusions regarding parental influence on safe sexual behavior for this age group. Thus, our study should be replicated with a larger sample, including a comparable number of sexually experienced and unexperienced participants of all ages of interest in order to satisfactorily determine intervention effects.

Lastly, we explored whether DPV’s delivery dose influenced the effects of the parental component at improving STI/HIV preventive behavior self-efficacy among participants in the experimental condition. The results showed a non-significant trend on the effect of dose, which indicated that, if tested with a larger sample, the parental intervention may be significantly effective at improving STI/HIV prevention self-efficacy for low and high dose interventions. This result is line with prior reports indicating that low and high dose interventions did not deliver significantly different outcomes [33]. There is yet to be support for the long-term effects of low and intensive methods of delivery [33].

Our findings should be interpreted considering potential limitations. First, due to limited parental availability, participants were not randomized and were assigned to a condition based on whether their parent received the intervention or not. Acknowledging this limitation, we examined the baseline differences of demographic variables between conditions and considered possible confounders when interpreting results. Although no sociodemographic differences between conditions were found due to non-random assignment, unknown confounders remain a threat. To extend the current findings, conducting an RCT with sufficient statistical power would be favorable to obtain a homogeneous distribution of participant characteristics throughout conditions, increase internal validity, and minimize the risk of working with biased groups [37]. Moreover, because we did not include any follow-up measures, this study might be too short timed to derive conclusions about the consistency of effects. Future investigations would benefit from including follow-up measures to identify the long-term effects of parental involvement in these programs. Furthermore, we administered paper-based self-report measures, which participants may have been reluctant or ashamed to answer truthfully. We controlled for this by ensuring the confidentiality of their reports. Based on findings suggesting that electronic methods of data-collection can improve sport-based HIV prevention program participant’s comfort and ensure their privacy [48], future studies can utilize online surveys for data collection. Finally, the dose of the parental workshop may have been low in relation to that of other related programs (e.g., [39]). Thus, research addressing the impact of an increased dose of the parental component would provide insights as to whether these interventions should require additional parental participation.

Despite these limitations, our findings present significant implications for theory and intervention and suggest that SCT provides a suitable theoretical approach for interventions targeting risky sexual behavior in youth of a cultural background characterized by a deficiency of resources. The present study validates the theoretical relevance of simultaneously promoting a protective familial environment (e.g., parents) in sport-based HIV prevention programs targeting youth. Thus, the inclusion of parents in these interventions can be an additional strategy to potentiate their effect on adolescent sexual behavior. Nonetheless, further research is needed to enhance the development of suitable interventions.

An important step for the future implementation of our intervention is to conduct further analyses to identify additional relevant needs in this population, form focus groups to reevaluate the design, and collect participant feedback to make relevant modifications [49]. Another direction for future research is to assess what parental figure participants refer to when answering the PTSRC-III, PMI and PRS, ensure that the parent mentioned is the one receiving the intervention, and examine the influence of participant’s and parent’s sex on the effect of protective parenting practices. The sex of the parent and the adolescent has been associated with the regularity and thoroughness in which parents apply protective practices on their children [47]. For instance, mothers are typically the primary communicators of sexual topics, whereas fathers appear to communicate about certain sexual topics (e.g., condom use) more often with their sons than with their daughters [50]. Distinguishing the sex of the parent and the adolescent will allow for an exhaustive analysis of possible disparities between mothers’ and fathers’ parenting practices and will guide the process of how to potentiate the impact of parental influence on youth behavioral outcomes.

## 5. Conclusions

This study on parental involvement in sports-based HIV prevention programs represents a novel mechanism to deter youth of resource-constrained communities from risky sexual behavior. We found encouraging trends suggesting that parental involvement in sports-based HIV prevention programs can enhance the positive effects of the program regarding increasing youth’s self-efficacy to practice HIV preventive behaviors by fostering appropriate parent–child communication about sex, parental monitoring, and a high parent–child relationship quality. Further research with a randomized design is needed, as well as future investigations into the long-term effects of parental involvement in these programs.

## Figures and Tables

**Table 1:** Descriptive statistics at baseline for total sample (*N* = 90).

Variable	UNICA (*n* = 42)		A Ganar (*n* = 48)	
	Control (*n* = 25)	Experimental (*n* = 17)	Control (*n* = 33)	Experimental (*n* = 15)
Mean Age (*SD*)	14.64 (±1.19)	15.29 (±1.21)	20.09 (±2.16)	19.13 (±1.13)
Percent Male	0	0	24.2	33.3
Percent Had Sex	16	17.6	87.9	73.3
Percent Education:				
9th–10th Grade	100	100	15.1	13.3
11th–12th Grade	0	0	21.2	60
High School Degree	0	0	60.6	26.7
Percent Born DR	100	100	84.8	100
Percent Born Haiti	0	0	15.2	0
Percent Speaks Spanish at Home	100	100	90.9	100
Percent Religion:				
Catholic	68	82.4	24.2	20
Cristian	24	17.6	57.6	66.7
None	8	0	18.2	13.3

Note. No significant differences between groups within intervention delivery program.

**Table 2:** Mean composite scores of UNICA and A Ganar for key outcomes and covariates.

	**Baseline DPV UNICA**		**Post DPV UNICA**	
Variable	Control (*SD*)	Experimental (*SD*)	Control (*SD*)	Experimental (*SD*)
SE STI/HIV	35.72 (± 6.01)	36.71 (± 4.61) ^c^	38.84 (± 5.51)	44.18 (± 4.79) ^c^
SSBQ	69.25 (± 4.99)	73.00 (± 8.72) ^a^	79.50 (± 4.80)	84.33 (± 2.52) ^a^
PTSRC-III	22.72 (± 5.98) ^a^	18.53 (± 7.00) ^c^	21.08 (± 5.31) ^a^	27.47 (± 5.58) ^c^
PMI	52.08 (± 15.28)	46.71 (± 17.32) ^c^	49.68 (± 16.22)	52.35 (± 15.00) ^b^
PRS	22.28 (± 2.61)	23.18 (± 1.13) ^b^	22.48 (± 2.49)	23.65 (± 2.61) ^b^
	**Baseline DPV A Ganar**		**Post DPV A Ganar**	
Variable	Control (*SD*)	Experimental (*SD*)	Control (*SD*)	Experimental (*SD*)
SE STI/HIV	36.39 (± 4.25)	30.53 (± 6.94) ^c^	38.03 (± 5.34)	40.53 (± 5.54) ^c^
SSBQ	70.97 (± 7.16)	73.33 (± 7.13) ^c^	74.76 (± 6.71)	84.50 (± 5.27) ^c^
PTSRC-III	21.76 (± 7.34) ^a^	22.67 (± 7.20) ^b^	20.15 (± 6.76) ^a^	28.40 (± 3.48) ^b^
PMI	47.03 (± 12.44)	53.73 (± 16.92)	52.42 (± 14.96)	54.60 (± 14.30)
PRS	19.18 (± 4.26)	18.07 (± 4.53) ^b^	18.94 (± 4.91)	21.53 (± 2.36) ^b^

Note. Significant differences between condition means in UNICA and in A Ganar. *SE STI/HIV* = Self-Efficacy for STIs and HIV Prevention Scale; *SSBQ* = Safe Sex Behavior Questionnaire; *PTSRC-III* = Parent–Teen Sexual Risk Communication Scale; *PMI* = Parental Monitoring Instrument; *PRS* = Positive Relationship with Parents Scale. ^a^ *p <* 0.05, ^b^ *p* < 0.01, ^c^ *p* < 0.001.

## Data Availability

The data presented in this study are available upon request from the corresponding author. The data are not publicly available due to privacy and ethical considerations.
